# Moderating Effect of the Power–Distance Belief on the Relationship between Employees’ Service Failures and Customers’ Behavioral Outcomes in the Sport Service Industry

**DOI:** 10.3390/ijerph18052488

**Published:** 2021-03-03

**Authors:** Hyunseok Song, Kevin K. Byon

**Affiliations:** Department of Kinesiology, Indiana University, Bloomington, IN 47405, USA; song1@indiana.edu

**Keywords:** fitness customer, power–distance belief, service failures, sport service

## Abstract

This study was designed to examine the moderating effects of the power–distance belief (PDB) on the relationship between employees’ service failures and customers’ transactional and non-transactional outcomes in a fitness center context. To test the relationships among these variables, we employed two pretests and a main experiment. In Pretest 1, a critical incident technique (CIT) was used to identify the employees’ service failure situations in fitness centers. Then, in Pretest 2, we developed two written scenarios that described employees’ service failures according to low and high severity and confirmed the differences between these two scenarios with a manipulation check. In the main experiment, we employed scenarios to examine the relationships among service failures’ severity, PDB, and customers’ non-transactional and transactional outcomes. We used Hayes’ PROCESS macro to test the PDB’s single moderating effect on the relationship between the service failures’ severity and the customers’ responses. According to the results, the moderating effect on the relationship between the service failures’ severity and fitness center customers’ non-transactional and transactional behaviors was confirmed. We extended the understanding of fitness center customers’ reactions, depending upon individual PDB to service failures, by comparing low- and high-service failure situations. Our findings also suggest that segmenting fitness center customers may help managers recognize that their customers’ varying responses depend on PDB.

## 1. Introduction

A gym management company report indicated that one of the most common problems in the fitness industry is attributable to gym owners’ poor customer service [1]. This report stated that without staff members who are knowledgeable about fitness and support customers’ exercises, customers lose their motivation to engage in fitness activities easily and rapidly and cancel their memberships. According to Briggs [2], the training staff’s attitude toward fitness centers (i.e., their skepticism and indifference to their gyms’ management) is the most critical roadblock to profitability. Thus, frontline employees who provide excellent service are the key to fitness centers’ success. However, employees’ services are not always perfect, and sometimes service failures are inevitable.

Once a service failure has occurred, customers respond with adverse behavioral outcomes that include, but are not limited to, negative word-of-mouth, switching behavior, and complaints to the service provider [3,4,5].

As service failures lead to customers’ unfavorable responses, the failures’ severity also affects these responses. Scholars have proven that more severe service failures tend to cause customers to express more hostile responses [5]. For example, when comparing a less severe service failure (e.g., delayed service) to a more severe service failure (e.g., canceled service), customers’ adverse responses increased after they experienced the latter failure [5].

Although service failures and their severity affect customers’ responses, customers do not always behave unfavorably when faced with service failures. This is because service providers have already prepared their responses to reasonable service failures. These efforts are referred to as service recoveries, and service providers’ prompt recovery efforts decrease customers’ adverse behaviors. Scholars have asserted that service providers’ efforts to compensate for their service failures do alleviate their customers’ unfavorable responses [3]. For example, a service provider’s apology and offer of monetary compensation for a service failure often softens the customer’s intention to engage in unfavorable behaviors [3]. However, this compensation is not always the best solution, because it raises costs and limits the paying provider’s ability to manage the business successfully.

Several scholars have focused on the intrinsic factors that moderate customers’ unfavorable responses to service failures and do not consume the service providers’ resources [6]. Researchers have suggested that customers’ tendencies depend on their individual beliefs, and consequently, these tendencies lead to diverse judgments of the services provided [7]. By understanding the customers’ intrinsic factors that moderate the relationship between service failures and their subsequent responses, service providers can allocate their resources better to address their service failures.

With respect to each individual’s belief, we focused on the moderating effects of power–distance belief (PDB) on customers’ reactions to service failures in a fitness center context, which refers to the degree of power disparity that customers accept in these inequalities in power [7,8,9,10]. A study showed that when a service provider delivered a low-quality service, some customers responded seriously to this problem. In contrast, others were not as concerned about the problem, depending upon their PDB. For example, customers who believe in the philosophy that “the customer is king” responded more unfavorably to low-quality service delivery. At the same time, other customers accepted the frontline employees’ efforts emotionally and denied the inequality in power between the service providers and themselves [9].

As for fitness center services, sport customers expect an excellent physical environment, high interactional quality, and a high-quality outcome [11,12]. Looking at the expected fitness center service quality, we examined the service failures in interactional quality for the following reasons. First, physical environments are being standardized, which is a global trend in all service industries, and thus, fitness centers’ physical environments are consistent with this trend. For example, in the U.S., popular fitness center franchises (e.g., Orangetheory Fitness, Anytime Fitness, and Planet Fitness) have over 1000 members each, and each franchisee provides a standardized physical environment. This standardization allows the owners to control their service delivery quality. However, with respect to outcome quality (e.g., energy, health, psychological wellbeing, and fitness) [11], the outcome does not originate with the service provider, but with the fitness centers’ customers.

On the other hand, interactional quality (e.g., trainers’ attitudes) is created between employees (e.g., trainers) and customers, and the intangible interactional quality is difficult to standardize [13]. In services related to personal interactions, customers’ evaluations are subjective, and this is ingrained in their intrinsic factors [6]. Scholars have claimed that PDB is a factor when customers evaluate services [9]. For example, Kim and Aggarwal [9] posited that customers with a high PDB expect a higher level of respect from a service provider than dothose with a low PDB.

Although PDB may provide a segmentation tool for customers, does it affect all service failures? For example, if a service provider causes a severe problem, do customers’ responses still vary? Depending upon the severity of the service failures, if customers’ responses are unified, they may handle the situation effectively. Further, previous scholars have suggested that excellent services solicit favorable responses from customers in the sport marketing context [14], yet few have investigated customers’ responses after they experienced service failures [4,6,15].

Previous studies have found the direct effect of service-related attributes on subsequent sport consumers’ responses, but they have not examined the way the moderating effects of the customers’ intrinsic factors may influence the direct effect. For example, a personal tendency to accept the social hierarchy may lead to customers’ expectation of a high level of employee courtesy, thus making the customers’ concern about employees’ discourteous behaviors a significant problem. To fill these research gaps, this study’s purpose was to identify specific service failure situations in fitness centers and examine PDB’s moderating effects between the service failure’s severity and the customers’ responses.

We suggest that fitness centers’ service is an ideal research topic for two reasons. First, in the fitness center industry, interactions and service processes with employees determine the fitness centers’ service quality with respect to the environment and programs [11,16]. Second, the three determinants of fitness centers’ service quality are the physical environment, physical activity program, and interactions with employees. These are pivotal factors that scholars have considered in their fitness center studies [11]. Among these three, environment and activity programs are standardized to reduce costs [11], but interactions with employees vary because they are based upon such human factors as employees’ courtesy [12]. In this competitive industry, employees’ service failures could result in considerable losses that affect a company’s ability to survive.

**Figure 1:**
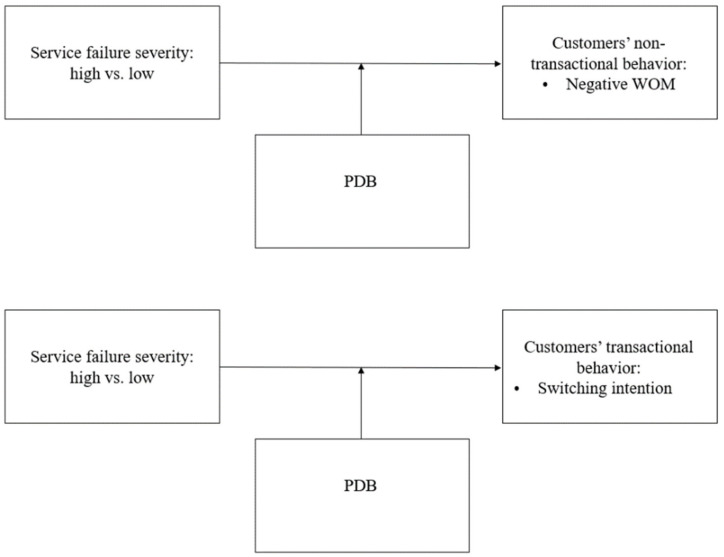
Research Model.

This study is organized as follows. We identified employees’ service failures in fitness centers. To do so, we employed the critical incident technique (CIT), a qualitative and exploratory method, to collect information by asking participants to recall remarkable incidents. Based upon these results, we developed service failure scenarios. We examined PDB’s moderating effects between the service failures’ severity and the customers’ unfavorable responses (i.e., non-transactional behaviors: negative word-of-mouth, and transactional behaviors: switching intention) [17]. We then present the results from our experiments and conclude by discussing their theoretical and practical implications. To examine PDB’s moderating effects on the relationship between employees’ service failure severity and fitness customers’ behavioral intentions, we followed Zhang et al.’s [10] individual PDB and tested its moderating effect on negative word-of-mouth and switching intentions when low- or high-severity service failures occurred in the fitness center industry (see Figure 1).

## 2. Literature Review and Hypotheses Development

### 2.1. Employee Service Failures in the Sport Industry

Since the 1990s, service researchers have often focused on service failures that result from a provider’s performance that did not fulfill the customer’s expectations [18]. Researchers have categorized service failures into three categories: (1) Employee responses to service delivery system failures (e.g., unavailability of service and unreasonably slow service); (2) employee responses to customers’ needs and requests (e.g., employees’ service failures related to customers’ direct requests), and (3) unprompted and unsolicited employee actions (e.g., employee’s attention paid to customer) [19]. Acknowledgments of service failures are beneficial to service providers. González et al. [20] found that service failure analysis and recovery management improved customers’ responses (e.g., satisfaction and retention) and financial performance (e.g., sales volume and growth, and profitability).

The imperfections in human nature give rise to the inevitability of employees’ service failures. We focused on employees’ responses and defined service failures as situations wherein their responses did not fulfill customers’ expectations. According to the service failure research, employees’ responses play a pivotal role in service failures [19,21]. For example, Groth and Grandey [22] proposed a conceptual framework rooted in the dual-perspective negative exchange spiral in the service interactions between employees and customers. When employees perceive customers’ mistreatment, then these employees are more likely to respond unfavorably (e.g., sabotage), and this increase in employees’ problematic behaviors also increases the likelihood that customers will react unfavorably (e.g., complaining) [22].

On the other hand, human resource management (HRM) scholars have focused on external factors that affected employees’ neglectful attitudes toward their tasks [23,24]. Scholars have associated employees’ withdrawal behaviors (e.g., an employee is working but not engaged) with their organizational performance (e.g., customers’ satisfaction) [23,24]. The authors emphasized that strategic HRM programs decreased employees’ withdrawal behaviors and increased organizational performance [23,24].

Although all employees work for their organization, some do not engage in organizational-related performance, as demonstrated by their products and services’ quality [23]. To reduce employees’ turnover intentions and improve their organizational performance with respect to service quality, scholars have suggested using human-resource-based training and practices such as strategic HRM and organizational leadership [23,24].

In the sport service industry, researchers have sought to acknowledge favorable and unfavorable services. Greenwell et al. [4] employed a CIT to identify spectators’ favorable and unfavorable experiences. They found that customers are most likely to complain about staff reliability and courtesy, both of which influence customers’ satisfaction with respect to employees’ interactions unfavorably. Among the various service industries, the fitness center industry depends on employees’ interactions, because frontline employees directly provide fitness center programs (e.g., personal training programs or group exercises), so sport management scholars have emphasized employees’ interactions with customers when they measured the quality of fitness services [11,16]. Chang and Chelladurai [25] suggested that the combination of service failure and recovery is an important dimension necessary to evaluate fitness services’ service quality overall. To date, few researchers have investigated customers’ responses to service failures in a sport context [6,26]. Consiglio and Van Osselaer [6] examined customers’ switching intention related to poor service in a fitness center setting, while Kim et al. [26] examined customers’ revisit intention related to their unfavorable experiences (i.e., other customers’ dysfunctional behaviors).

### 2.2. Customers’ Behavioral Outcomes after Employees’ Service Failures

When firms’ services do not fulfill customers’ expectations during their service encounters, many customers react unfavorably to these problematic situations. When service employees provide poor services, customers perceive unfairness and injustice, and they engage subsequently in unfavorable responses [27]. Scholars have categorized customers’ behaviors that service providers affect in two ways: Non-transactional and transactional behaviors [17,28,29]. Non-transactional customer behaviors refer to non-cost-based responses to service experiences. Customers evaluate and describe their service experiences to other current or potential customers. When customers experience service failures, they communicate their unfavorable evaluations to other customers, and the common non-transactional customer behavioral outcome is negative word-of-mouth [28].

On the other hand, transactional customers’ behaviors refer to their cost-based responses to service experiences. In the relationship between customers and service providers, customers pay the costs, and the service employees provide the benefits corresponding to the customers’ payments. However, customers who have experienced service failures consider stopping their purchase of poor services and changing to an alternative service provider. The common transactional customer behavioral outcome in service studies is switching intention [29].

#### 2.2.1. Negative Word-of-Mouth

Negative word-of-mouth refers to the act of sharing experiences with problematic employees’ service with others through online or offline networks. Extant studies in service industry contexts have found that service failures influence consumers’ behavioral intentions to blame service failures and convey negative word-of-mouth directly [30]. In addition, customers who experienced a higher service failure severity were less inclined to recommend their service provider to others [5].

In the sport management context, Yu et al. [31] indicated that fitness center customers who experience low service quality, which includes employee-based service quality, are apt to share their bad experiences with others, thereby influencing potential customers, such as friends and relatives. Thus, high employee service failure severity may increase negative word-of-mouth and decrease positive comments.

#### 2.2.2. Switching Intention

Switching intention refers to customers’ intention to leave their original service provider in favor of another [29,32]. Research has suggested that the costs associated with new customer acquisitions are higher than those associated with retaining current customers, and the difference between the acquisition and retention costs is even more significant when they have numerous competitors [33].

Employees’ service failures are a major antecedent to customers’ switching behavior [6,32]. Of the eight antecedents of customers’ switching behavior—pricing, inconvenience, core service failure, service encounter failures, response to service failure, competition, ethical problems, and involuntary switching—Keaveney [32] determined that employees’ service encounter failures represented the second most critical incidents. When employees’ service failures are severe, customers may be more inclined to sever ties with their original service provider.

### 2.3. Service Failure Severity’s Effect on Customers’ Behavioral Outcomes

Service failure severity refers to the magnitude or intensity of a given failure [5]. For example, in the airline industry, a scheduled flight’s cancellation is a more severe failure than is a delayed departure [5]. When service failure severity is high (e.g., the potential cost to address service problems exceeds $100), 19% of consumers still intend to do business with the offender, even if the initial problem is not resolved [34]. However, 54% of these consumers still intend to do business with the offender when the problem is resolved. When service failure severity is low (e.g., the potential cost to address service problems is from $1 to $5), 46% of consumers still intend to do business with the offender when the problem is not resolved. Still, 70% of consumers are willing to do business with the offender when the problem is resolved [34]. Thus, the likelihood that consumers perceive a loss and complain about a situation depends upon the service failure’s severity.

Researchers have found that, among diverse service industries (e.g., restaurants and airlines), the higher a service failure’s severity, the greater the consumers’ unfavorable reactions [5,30]. In the sport consumer behavioral context, Kim et al. [35] tested customers’ responses to the magnitude of given service failure situations on the part of an airline company that sponsors sport teams (flight cancelations vs. delays) and the way they affected sport consumers’ repurchase intention.

Although the severity of service failures attributable to employees in fitness centers has not been the main focus of previous literature, we expect these failures’ magnitude to be applicable to fitness center services because they prompt customers’ unfavorable behavioral outcomes in other industries (e.g., airline ticket services) [5].

### 2.4. Moderating Effects of Power–Distance Belief (PDB)

Scholars have proven that service failures severity increases customers’ negative responses [5,30]. While service failure severity is an important extrinsic factor in customers’ unfavorable responses in the cognitive-emotive process, personal characteristics are also critical antecedents [36]. Although customers experience the same severity of employees’ service failure, each may respond differently depending upon his/her personal background.

Scholars have focused on PDB’s effects on customers’ consumption behaviors [7,8,37]. According to the level of PDB, individuals’ acceptance of others’ opinions or attitudes varies [38]. For example, an individual who is considered to have a high PDB may believe that people should put a higher value on conformity as citizens. In contrast, an individual who is considered to have a low PDB may believe that disagreeing with the boss (e.g., employer, manager, or supervisor) will increase productivity [10]. Scholars have also found that PDB moderates the effect between service providers and customers [9,39,40]. For example, Song et al. [39] posited that compared to those with a high PDB, customers with a low PDB preferred user-designed products to designer-designed products. These low-PDB customers were more likely to prefer engaging in value co-creation and making a decision in non-authoritarian environments [39]. Customers’ PDB also plays a role among the social relationships.

With restaurant-related scenarios, Kim and Aggarwal [9] examined the way customers in a culture with a higher PDB level (i.e., Eastern culture) expect a higher level of respect and courtesy from their service providers at the country level (i.e., Canada and South Korea). The authors found that customers in the high-PDB culture accepted the hierarchy between service providers and customers, and this perception caused them to expect employees to be more courteous. Recently, Xu et al. [40] reported that customers’ sympathy depends on PDB related to humans rather than companies. The authors also found that low PDB-customers tended to empathize with the victims of companies’ moral transgressions because they are more likely to empathize with less powerful, vulnerable individuals than powerful, dominating organizations [40].

Customers’ power is increasing in competitive service markets, and the slogan “the customer is king” has re-emerged; subsequently, customers have used their power when explaining their favorable or unfavorable responses to service failures [9]. The question is, which customers will respond more unfavorably to service failures according to their PDB level? Assuming that customers’ power levels are higher than those of service employees, previous scholars have claimed that customers in high-PDB cultures (e.g., Eastern cultures) expect a higher level of service quality and are more likely to complain about service failures compared to those in low-PDB cultures (e.g., Western cultures) [9]. Thus, we expect PDB to moderate the relationship between the service failures’ severity and customers’ unfavorable responses positively in a fitness center program.

**Hypothesis** **1.** *Compared to customers with a low PDB, customers with a high PDB will respond more unfavorably to employees’ service failures in a fitness center program.*

**Hypothesis** **1a.** *Compared to customers with a low PDB, customers with a high PDB will be more likely to engage in unfavorable non-transactional behaviors in response to employees’ service failures in a fitness center program.*

**Hypothesis** **1b.** *Compared to customers with a low PDB, customers with a high PDB will be more likely to engage in transactional behaviors in response to employees’ service failures in a fitness center program.*

Employees’ service failure situations are not homogeneous [1]. Previous scholars have classified service failures according to their severity [5,41]. Not surprisingly, customers who experienced a severe level of employees’ service failures engaged in more unfavorable responses than did those who experienced a moderate level of service failures [5,41]. However, previous scholars have reported controversial results of PDB’s moderating effect on the relationship between provider-caused problematic situations and customers’ subsequent behaviors [7,9,10,40]. For example, several scholars [7,9,10] have claimed that customers with a high PDB reacted more unfavorably to service failures, while Xu et al. [40] asserted that customers with a low PDB responded more actively to companies’ moral transgressions and were more empathic toward the victims.

We assume that the PDB effect varies depending upon the level of the service failure severity. Although previous scholars have examined problematic situations service providers caused, each situation’s severity varied. For example, Lalwani and Forcum [7] examined PDB’s moderating effect on a dollar amount loss attributable to a service failure and found that high-PDB customers were more concerned about the small loss than were the low-PDB customers. On the other hand, Xu et al. [40] tested PDB’s moderating effect on a company’s serious gross fault (i.e., violating employee rights and welfare), which caused a massive number of victims. They found that low-PDB customers reacted more aggressively to a company’s fault because they had more empathy for the victims.

Thus, we questioned when PDB’s moderating effect is noticeable by comparing PDB’s moderating effect on low- and high-severity service failures. The slope of the value function of the proposed theory suggests that the difference in customers’ perceived loss among other individuals is smaller when customers perceive that the loss is severe rather than moderate [42]. Personal perception of a loss is less sensitive when the loss is more severe [42]. Thus, we assumed that PDB’s moderating effect is smaller in severe service failures. On the other hand, its moderating effect will be larger in service providers’ minor mistakes. Therefore, we propose the following hypotheses:

**Hypothesis** **2.** *PDB’s moderating effect will be smaller with a higher level of service failure severity than a lower level of service failure severity.*

**Hypothesis** **2a.** *PDB’s moderating effect on the relationship between service failure severity and negative word-of-mouth will be smaller with a higher level of service failure severity than a lower level of service failure severity.*

**Hypothesis** **2b.** *PDB’s moderating effect on the relationship between service failure severity and switching intention will be smaller with a higher level of service failure severity than a lower level of service failure severity.*

## 3. Methods

To test our research hypotheses, we conducted a total of three studies, Pretests 1 and 2, and a main experiment. Specifically, we performed two pretests to identify the severity of service failures in the fitness industry and developed experimental survey scenarios: Pretest 1: Selection of the Stimuli and Pretest 2: Manipulation Check.

### 3.1. Pretest 1: Selection of the Stimuli

We investigated specific service failure cases before we developed an experimental design with written scenarios related to employees’ service failures in fitness centers. Although sport marketing scholars have focused on fitness center service studies, specific service failure situations in fitness center programs have not been identified. Thus, to identify frequently occurring service failures, we conducted a study using the critical incident technique (CIT), a qualitative and exploratory method designed to collect information about specific incidents according to individuals’ memories of those incidents. CIT is the proper method to explore customers’ experiences with service failures [4].

First, the respondents were asked two screening questions: (1) “Are you currently a fitness center member?” and (2) “Have you experienced dissatisfying service encounters that were caused by a fitness center employee?” Respondents who confirmed both questions were asked to recall specific problematic events employees caused. We modified Bitner et al.’s [19] open-ended questions to identify the particular events and related employee behaviors that caused employee service failures in the fitness center industry: (1) “What specific event led to a dissatisfying service encounter?” and (2) “What did the contact employee do that caused these events to be remembered with distaste?” For Pretest 1, we recruited 100 participants via Amazon’s Mechanical Turk (MTurk), which is a crowd-sourcing website to hire online survey participants remotely for a certain amount of money. These 100 current or former fitness center members had experienced service failures at a fitness center (45% males; mean age = 36.4 years; minimum age = 20; maximum age = 68). According to the International Health, Racquet & Sportsclub Association’s (IHRSA) 2018 global report, the average fitness customer’s age is approximately 40 years, and most members are 25 through 60. Hence, our sample’s demographics were similar to a wide range of global fitness center statistics [43].

We identified multiple types of service failures (see Table 1). Among the total service failures, neglecting to offer assistance (28%), rudeness (23%), ignorance (15%), confrontation (11%), and shaming the customer (7%) occurred most frequently. These results were largely consistent with Consiglio and Van Osselaer’s [6] poor service scenario in a fitness center program. In their scenario, the employee (i.e., trainer) caused many service failures included in the Pretest 1 results. These failures included neglecting to offer assistance/ignorance (e.g., “He is not attentive” and “He does not really show interest in you as a person”), and rudeness/shaming the customer (“He points out your flaws”).

While Consiglio and Van Osselaer [6] provided a poor service situation, this study compared two different levels of service failures with respect to severity. We modified Consiglio and Van Osselaer’s [6] poor service failure scenario to develop low- and high-severity employee service failures. We developed these two scenarios for the degree of the employee’s poor service failure by following Chelminski and Coulter’s [41] service failure severity study. They suggested written scenarios of low- and high-severity employee service failures in general business environments (i.e., retail and repair services). According to their scenarios, the employees’ verbal and non-verbal responses caused concern among customers when they perceived the severity of the level of service failure.

The Pretest 1 results indicated that fitness center customers are concerned about the multiple types of employees’ problematic services that correspond with previous studies’ scenarios [6,41]. Based upon extant studies and the Pretest 1 results, the two scenarios developed reflected high employee service failure severity, and low employee service failure severity.

#### 3.1.1. Low Employee Service Failure Severity Scenario in Fitness Center Programs

Imagine that you are in a personal training program or a group exercise class. Your trainer has very good credentials and does the job but is not particularly attentive. The trainer is nice, but sometimes does not look interested in you as a person. The trainer points out your qualities from time to time but does not usually provide positive feedback or offer encouragement.

#### 3.1.2. High Employee Service Failure Severity Scenario in Fitness Center Programs

Imagine that you are in a personal training program or a group exercise class. Your trainer has very good credentials and does the job but is not attentive at all. The trainer is not nice and does not show any interest in you as a person. The trainer frequently points out your flaws, but never provides positive feedback or encouragement.

### 3.2. Pretest 2: Manipulation Check

In Pretest 2, we conducted a manipulation check for these two scenarios. First, to limit respondents to those who were currently fitness center program members, they were asked to answer the following screening question: “Are you currently a fitness center program member?” Upon confirmation of their eligibility, a total of 55 respondents was recruited via MTurk and assigned randomly to one of the two scenarios (*n* = 26 for low severity; *n* = 29 for high severity). Next, as a manipulation check, we adopted Kim et al.’s [35] service failure severity scale. The respondents were asked to answer three items about the severity of service failures on a 7-point Likert scale (α = 0.96). We asked: If this problem were really happening to me, (1) would I consider the problem to be very severe? (2) would I consider it as a big problem, or (3) would it make me feel very angry? The result of the manipulation check for the service failure severity showed a significant difference between the two severity scenarios (*M*_low severity_ = 3.97 vs. *M*_high severity_ = 5.17, *t* = −2.63, *p* < 0.05). Therefore, the manipulation check was successful.

### 3.3. Main Experiment

#### 3.3.1. Sample and Design

To examine PDB’s moderating effects on the relationship between an employee’s service failure severity and the customer’s responses (i.e., non-transactional behavior and transactional behavior), we used a 2 × 2 between-subject experimental design based upon the written scenarios (high vs. low service failure severity × high vs. low PDB). Following previous studies [17,28], we measured the non-transactional behavior as negative word-of-mouth and the transactional behavior as switching intention.

This study’s target population included current and previous fitness center members who had attended physical activity classes. At the beginning of the survey, screening questions were asked to identify whether the respondents had actually participated in fitness center programs. According to Hair et al.’s [44] suggestion for the sample size for multiple regression and using G-Power analysis with three predictors and 0.8 power, we decided that a sample size larger than 100 was appropriate for the study. Further, most previous studies that used a mediation analysis used a sample size ranging between 100 and 350 [45].

Ultimately, we recruited a total of 250 U.S. adult consumers via MTurk. To increase data quality, we limited participants to those with the following records: 97% or higher acceptance rate of their responses, and 500 or more experiences completed on MTurk [46]. Peer et al.’s [46] results indicated that MTurk is a crowd-sourced participant pool applied to various social scientists’ research, and it uses quality options to provide sufficient conditions for data quality. Of these 250 respondents, six were eliminated because of incomplete survey questionnaires. Thus, 244 respondents were used in the subsequent data analyses (60% males; average age = 34.6 years; minimum age = 20; maximum age = 70; ethnicities: Caucasian = 77%, African American = 10%, Asian = 7%, Hispanic = 5%, Other = 1%; education levels: Associate graduate = 30%, Bachelor’s degree = 59%, Master’s degree = 11%).

#### 3.3.2. Measures

The same items of service failures’ severity as measured in Pretest 2 were used (*α* = 0.94). PDB was considered both a cultural level and individual level construct [10]. We adapted the PDB scale for the individual level. Thus, PDB was measured using Zhang et al.’s [10] scale of eight survey items (e.g., “As citizens, we should put a high value on conformity”; 1 = strongly disagree, 7 = strongly agree; *α* = 0.74). As Bougie et al. assessed both transactional and non-transactional behaviors, which correspond with our study’s context, we adapted their scale [28]. Specifically, two items were used to measure negative word-of-mouth (e.g., “Say negative things about the service provider to other people”; 1 = strongly disagree, 7 = strongly agree; *α* = 0.85). Switching intention was measured using six items adapted from Bougie et al.’s scale [28] (e.g., “I will not use this service in the future”; 1 = strongly disagree, 7 = strongly agree; *α* = 0.96).

#### 3.3.3. Procedures

Participants were assigned randomly to one of the two conditions (i.e., service failure severity: Low vs. high). Initially, they were asked to read a scenario that described an employee’s service failure situation during a fitness center program. To manipulate the failure’s severity level, participants were provided with two situations that described high- and low- service failure severity. After reading one of these two scenarios, participants answered the questionnaire assigned, which asked questions meant to gauge PDB and customers’ negative word-of-mouth. Data normality was checked by assessing skewness and kurtosis. The thresholds for skewness and kurtosis were between −3 and +3 [44]. These findings revealed that all skewness and kurtosis values were within the threshold (i.e., between −2 and 2).

## 4. Results

### 4.1. Manipulation Check

A *t*-test revealed a significant group difference (low service failure severity: *n* = 120, *M_low severity_* = 3.62, *SD_low severity_* = 1.64 vs. high service failure severity: *n* = 124, *M_high severity_* = 5.12, *SD_high severity_* = 1.38, *t* = −7.54, *p* < 0.01), indicating that people perceived the service failure to be more severe in the high-service failure severity scenario.

### 4.2. Construct Validity

Confirmatory factor analysis (CFA) was conducted to assess the survey items’ validity. All six items for the constructs were included in the measurement model, and the model fit statistics overall were acceptable (χ^2^ = 12.4, df = 8, CFI = 0.99, TLI = 0.98, RMSEA = 0.05, and SRMR = 0.03). CR values were employed to measure the scales’ reliability. Per Hair et al.’s [44] suggestion, the study model was compared with a 0.70 cutoff value, and all CR values were higher than 0.70 (PDB = 0.73, negative word-of-mouth = 0.85, switching intention = 0.95). Factor loading significance was employed to determine convergent validity. All of the factor loadings were greater than 0.50 and were consistent with Hair et al.’s [44] recommended cutoff value.

Discriminant validity was examined via a comparison between the squared correlation values and AVE estimates. When the squared correlation values are lower than the AVE estimates, discriminant validity is supported [47]. The inter-factor correlation between PDB and negative word-of-mouth was 0.104, and the inter-factor correlation between PDB and switching intentions was 0.164, respectively. In addition, no value of the squared correlation exceeded the AVE estimates. Thus, this model satisfied discriminant validity.

### 4.3. Hypothesis Testing

To examine the hypotheses, we used Hayes’ PROCESS macro [48]. The PROCESS macro (Version 3.4, Columbus, OH, USA) is a statistical package used to compute path analysis-based moderation and mediation analysis. The PROCESS macro provides approximately 100 types of moderation and mediation models to compute paths. We applied Model 1 of the PROCESS macro (Model 1; 5000 bootstrap resampling) corresponding with this study to test PDB’s single moderating effect on the relationship between service failure severity and negative word-of-mouth [48]. Moreover, we used the PROCESS macro to conduct a simple slope analysis that allowed us to compare the groups’ differences according to the PDB levels (high vs. low). PDB was centered as the mean equal to zero, and service failure severity was coded −0.5 for the low severity and 0.5 for the high severity, respectively.

PDB’s moderating effects were detected in the relationship between the service failure severity and negative word-of-mouth (*F*(1, 243) = 3.92, *p* < 0.05) (see Table 2). When service failure conditions were controlled, PDB increased customers’ negative word-of-mouth (*b* = 0.28, *SE* = 0.11, *p* < 0.05, 95% CI = 0.07 to 0.50). Thus, H1a was supported. Further, PDB played a moderating role between service failure severity and switching intention (*F*(1, 243) = 3.93, *p* < 0.05) (see Table 3), and the PDB level affected customers’ switching intention positively (*b* = 0.31, *SE* = 0.10, *p* < 0.05, 95% CI = 0.11 to 0.51). Thus, H1b was supported.

**Figure 2:**
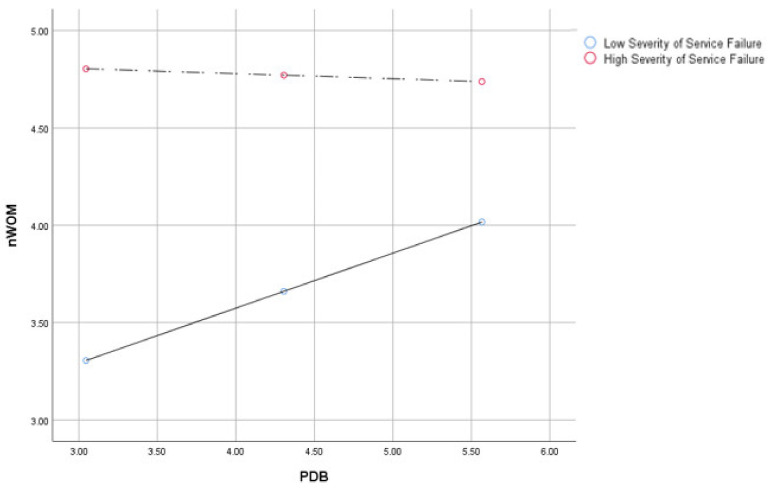
PDB’s moderating effect on the relationship between service failure severity and negative word-of-mouth.

**Figure 3:**
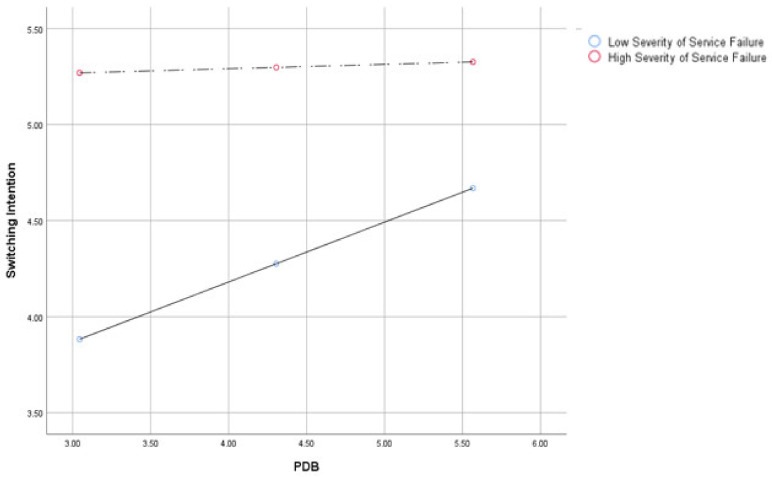
PDB’s moderating effect on the relationship between service failure severity and switching intention.

When we investigated the PDB effects according to the severity of service failure situations, the effect was larger (*b* = 0.28, *SE* = 0.11, *p* < 0.05, 95% CI = 0.07 to 0.50) than in the high severity service failure situations (*b* = −0.03, *SE* = 0.11, *p* > 0.05, 95% CI = −0.24 to 0.19) associated with negative word-of-mouth. Similarly, the PDB effect was larger for switching intention in the low service failure severity (*b* = 0.31, *SE* = 0.10, *p* < 0.05, 95% CI = 0.11 to 0.51) than in the high service failure severity (*b* = 0.02, *SE* = 0.10, *p* > 0.05, 95% CI = −0.18 to 0.23) (see Figure 2 and Figure 3). Thus, H2a and b were supported.

## 5. Discussion

To examine PDB’s moderating effect on fitness customers’ transactional and non-transactional behaviors when they encounter an employee’s service failure, we identified specific service failures that occurred frequently in fitness centers. Then, we tested customers’ transactional and non-transactional behavioral intentions by applying an experiment that used written scenarios. The findings indicated that (1) customers with high PDB engaged more in unfavorable behaviors, such as negative word-of-mouth and switching intention compared with customers with low PDB and (2) PDB’s moderating effect was smaller when customers experienced severe service failure situations. Overall, this study’s results have several theoretical and practical implications, which are discussed in the following sections.

### 5.1. Theoretical Implications

Scholars have recognized PDB’s role in affecting customers’ attitudes and behaviors after they evaluated frontline employees’ poor service quality [9]. Kim and Aggarwal [9] found a difference in high- and low-PDB customers’ responses to frontline employees’ service quality at the cultural level by comparing Western customers (low PDB) to Eastern customers (high PDB). We echoed their results and extended them to the individual level. When fitness customers experienced an employee’s problematic services, those in the high-PDB group engaged in more negative word-of-mouth and had a greater switching intention than did those in the low-PDB group.

While previous studies have proven PDB’s effects on the service furnished in a one-time experience (e.g., food court service [9], we identified PDB effects in a periodic service: Fitness programs. Further, we extended the understanding of fitness center customers’ reactions to service failures by comparing low- and high-service failure situations. According to Kahneman and Tversky’s prospect theory, [42], the slope of the theory’s value function suggested that the difference in customers’ perceived loss is smaller when they perceive that it is a severe loss rather than a moderate loss. With respect to the magnitude of service failure severity, PDB’s moderating effect was larger in the low severity than the high severity service failures. Thus, Kahneman and Tversky’s value function was indeed found to be applicable in explaining fitness center customers’ reactions to service failures. We also found that customers with high PDB engaged more in both non-transactional and transactional behavioral intentions compared to those with low PDB. With respect to supporting Yoshida et al.’s fan engagement framework, [17], we extended and specified fitness customers’ non-transactional and transactional behavioral intentions toward service failures depending upon their PDB level.

### 5.2. Practical Implications

This study also has significant practical implications for PDB’s moderating effect on the relationship between service failure severity and fitness customers’ non-transactional and transactional behavioral intentions.

First, we investigated individuals’ service failure experiences attributable to fitness center employees’ inappropriate services by applying CIT. Customers indicated that employees neglected to offer assistance, were rude, showed ignorance, chose to be confrontational, and shamed customers in public frequently after the customers joined a fitness program. Thus, when fitness center managers interview candidates for a physical program instructor position, they should avoid the potential problems shown in these CIT results.

Second, when managers understand frontline employees’ problematic behaviors, they may prevent these problems before a service failure occurs. Fitness center customers recognize employees’ unfavorable behaviors clearly, and strategic HRM and leadership programs influence behaviors that cause service failures. Specifically, strategic HRM programs include the retention configuration of HR practices (i.e., making employees believe “my organization selects employees who believe in our organization’s core values”), acquisition/selection configuration (i.e., making employees feel “many employees in this organization are involved in the selection decision”), customer-service-related training, and internal employees’ availability (i.e., making employees feel “the salaries in my organization are competitive for this industry) [23]. By applying strategic HRM in employees’ education, their inappropriate behaviors toward customers may decrease.

Third, although managers make an effort to prevent service failures, they occur inevitably, and recovering from them is the manager’s responsibility. Our findings suggested that segmenting fitness center customers according to PDB may help managers recognize their customers’ various responses. These managers can collect PDB information when customers fill out their membership cards.

When severe service failures occur, managers can use a service recovery plan without segmenting customers into the rapid response category. For minor service failures, the low-PDB group’s customers responded more generously than did those in the high-PDB group. This indicates that the high-PDB group is more sensitive about minor service failures [7]. Thus, when a minor service failure occurs, fitness center managers can take care of their target customers and their complaints promptly. With this action plan, managers can apply their limited resources more effectively.

### 5.3. Limitations and Suggestions for Future Studies

This study’s limitations should be acknowledged. The first relates to the realistic nature of the service failure scenarios we developed. We applied the Pretest 1 results and previous study scenarios to develop our scenarios by compounding the multiple dimensions of service failures (e.g., neglecting to offer assistance, rudeness, ignorance, and shaming). Although our scenarios covered various feasible factors that cause service failures, they were able to examine only each factor’s direct effect on customers’ behaviors. Depending upon the individual, the prioritization of a situation’s severity may differ. Future research should employ multiple scenarios that are mutually exclusive contexts (e.g., frontline employees neglecting to help customers, rude employees, and employees laughing at customers) to measure sophisticated situations in each dimension.

Although we tested PDB’s moderating effect on the relationship between service failures and customers’ behaviors with multiple dimensions (i.e., non-transactional and transactional), this limitation of the study is related to the ability to generalize the findings. The service failure situations in the study were assumed in certain detailed situations, such as physical activity classes. Previous scholars have posited that fitness center customers’ concerns include broad factors (i.e., facility presentation, core services, staff, and parking [49]. To address this limitation, we need to examine additional service failures in fitness centers and expand our understanding of customers’ unfavorable reactions in broader cases.

## 6. Conclusions

Previous scholars have found that employees’ service failures cause fitness customers’ unfavorable responses with respect to transactional and non-transactional behaviors. We extended fitness consumer-related service failures studies by investigating PDB’s moderating effect. We examined its moderating effect on fitness center consumers’ transactional and non-transactional behaviors when they experienced service failures with a low or high level of severity. To examine the moderating effect, (1) we investigated feasible service failure situations using CIT, (2) developed written scenario describing low- and high-severity fitness center service failures, and (3) then conducted an experiment to examine fitness center consumers’ unfavorable responses depending upon their PDB level. A total of 244 individuals recruited via MTurk participated in the experiment. Our findings extended the understanding of fitness center customers’ heterogeneous responses, depending upon their intrinsic PDB and extrinsic service failure situations. The results of this study suggested that individual intrinsic values, such as PDB, moderate service failures’ direct effect on fitness consumers’ subsequent unfavorable responses. Moreover, we recommend that practitioners prevent employees’ service failure situations before they occur by developing strategic HRM and leadership programs and ways to respond to service failure situations by allocating available resources based upon the situation’s priority.

## Figures and Tables

**Table 1:** CIT Results of Pretest 1.

Typology	Frequency	Percentage	Sample Statement
Neglecting to Offer Assistance	28	28%	“A unit I wanted to use had a broken chain. It took 20 min for someone to be available to fix the issue.”
Rudeness	23	23%	“The assistant rudely attended to me by mentioning that the temperature could not be increased since another client had asked for the temperature to fall below the normal required, so I made a formal complaint to emphasize my first complaint.”
Ignorance	15	15%	“They wouldn’t even listen to me and told me just to go use something else or go for a walk.”
Confrontation	11	11%	“An employee started yelling at me because I didn’t do the exercise the right way.”
Shaming the customer	7	7%	“When I went to join the gym, they made me weigh in as part of the orientation and then suggested that I lose an unhealthy amount of weight. I am a normal weight and in very good shape.”
Others (e.g., excessive sales promotion, overcharging, inconvenient cancellation)	16	16%	“When I was interested in signing up for a membership, I was matched with a sales consultant who proceeded to waste a lot of my time by talking about how great the gym was.”

**Table 2:** Effects of Service Failure Severity and PDB on Negative Word-of-Mouth.

Variables	Variable	*B*	*SE*	*t* Value	*p* Value	95% CI
SF severity (IV)	nWOM (DV)	1.10	0.20	5.60	0.00	0.71 to 1.49
PDB (M)	nWOM (DV)	0.13	0.08	1.61	0.11	−0.03 to 0.29
SF severity × PDB (INT)	nWOM (DV)	−0.33	0.16	−2.05	0.04	−0.64 to −0.01

Note: SE = standard error; CI = confidence interval; SF = service failure IV = independent variable, nWOM = negative word-of-mouth; DV = dependent variable; PDB = power–distance belief; *M* = moderator; *INT* = interaction.

**Table 3:** Effects of SF Severity and PDB to Switching Intention.

Predictor Variable	Dependent Variable	*B*	*SE*	*t* Value	*p* Value	95% CI
SF severity (IV)	Switching Intention (DV)	1.03	0.18	5.60	0.00	0.67 to 1.40
PDB (M)	Switching Intention (DV)	0.19	0.07	2.51	0.01	0.04 to 0.34
SF severity × PDB (INT)	Switching Intention (DV)	−0.34	0.15	−2.27	0.02	−0.63 to −0.04

Note: SE = standard error; CI = confidence interval; SF = service failure IV = independent variable, DV = dependent variable; PDB = power–distance belief; *M* = moderator; *INT* = interaction.

## Data Availability

The data presented in this study are available on request from the corresponding author.
